# Identification of a Chemotherapeutic Lead Molecule for the Potential Disruption of the FAM72A-UNG2 Interaction to Interfere with Genome Stability, Centromere Formation, and Genome Editing

**DOI:** 10.3390/cancers13225870

**Published:** 2021-11-22

**Authors:** Senthil Renganathan, Subrata Pramanik, Rajasekaran Ekambaram, Arne Kutzner, Pok-Son Kim, Klaus Heese

**Affiliations:** 1 Department of Bioinformatics, Marudupandiyar College, Thanjavur 613403, India; rsenthil@mpi.edu.in; 2 Department of Biology, Life Science Centre, School of Science and Technology, Örebro University, 701-82 Örebro, Sweden; subrata.pramanik@oru.se; 3 Department of Chemistry, V.S.B. Engineering College, Karur 639111, India; ekambaramrajasekaran@gmail.com; 4 Department of Information Systems, College of Engineering, Hanyang University, Seoul 133-791, Korea; kutzner@hanyang.ac.kr; 5 Department of Information Security, Cryptology, and Mathematics, Kookmin University, Seoul 136-702, Korea; pskim@kookmin.ac.kr; 6 Graduate School of Biomedical Science and Engineering, Hanyang University, Seoul 133-791, Korea

**Keywords:** cell cycle, centromere, DNA repair, proliferation

## Abstract

**Simple Summary:**

Pivotal factors that contribute to tumorigenesis were subjected to analysis by molecular modeling. In particular, the FAM72A-UNG2 protein–protein interaction was modeled to predict a potential solution for the treatment of cancer. We screened chemical libraries to identify withaferin B as a lead molecule capable of interfering with the FAM72A-UNG2 interaction, thus opening new therapeutic avenues for cancer.

**Abstract:**

Family with sequence similarity 72 A (FAM72A) is a pivotal mitosis-promoting factor that is highly expressed in various types of cancer. FAM72A interacts with the uracil-DNA glycosylase UNG2 to prevent mutagenesis by eliminating uracil from DNA molecules through cleaving the N-glycosylic bond and initiating the base excision repair pathway, thus maintaining genome integrity. In the present study, we determined a specific FAM72A-UNG2 heterodimer protein interaction using molecular docking and dynamics. In addition, through in silico screening, we identified withaferin B as a molecule that can specifically prevent the FAM72A-UNG2 interaction by blocking its cell signaling pathways. Our results provide an excellent basis for possible therapeutic approaches in the clinical treatment of cancer.

## 1. Introduction

Genomic uracil bases may occur from cytosine deamination or the misincorporation of dUMP residues during DNA replication [1]. The uracil-DNA glycosylase UNG physiologically functions in the base excision repair (BER) mechanism of the cell in order to replace uracil from U/G mispairs with cytosine, thus preventing genomic mutations [2,3,4,5,6]. It excises unwanted genomic uracil bases using an extrahelical base recognition mechanism, thus preventing possible C-to-T transition mutations that eventually arise from cytosine deamination [7,8,9]. The resulting apurinic/apyrimidinic site (AP-site) is considered one of the most common DNA lesions in the genome, and a persistent AP-site can have adverse consequences, as the lesion disrupts many DNA and RNA transactions and leads to cytotoxic strand breaks, mutations, and other forms of genomic instability [1,10,11]. 

Human UNG exists in two different isoforms, mitochondrial UNG1 and nuclear UNG2, that are both encoded from the same single 13.5-kb nuclear UNG gene as a result of two separate promoters and alternative splicing [12,13,14]. While UNG1 and UNG2 share a common conserved catalytic domain, they contain differing N-termini sequences responsible for differential subcellular localization. Amino acid (AA) residues 1–92 make up the N-terminus of UNG2; they contain a nuclear localization signal of positively charged residue clusters (K and R residues), rendering UNG2 as the primary uracil-DNA glycosylase enzyme in the nucleus [14,15]. Interestingly, in the absence of binding partners, the N-terminal region is, for the most part, without a fixed structure [2,16,17,18]. UNG2 is rapidly recruited to sites of DNA damage where its N-terminus can interact with its catalytic site (which binds to uracil) and with chromatin. UNG2 colocalizes with CENP-A at centromeres and other sites of DNA damage in proliferating cells, thus implying that it is also required for chromosome segregation during mitosis [19].

Family with sequence similarity 72 A (FAM72A) is a novel gene expressed in the brain hippocampus area in proliferating neural stem cells, particularly during the G2/M-phase of the cell cycle [20,21]. Most strikingly, humans have four paralogs (FAM72 A-D), whereas all other species express just one ortholog [22,23]. Under pathophysiological conditions, FAM72A is also expressed in various proliferating cancer cells [24,25]. Notably, FAM72A interacts with UNG2 [26,27]. This denotes that the cellular role of FAM72A is as a cooperative partner for genomic BER in order to ensure genome integrity and impede the formation of cancer. Recent data show that decreased levels of FAM72A lead to hyperphysiological UNG2 levels, an increased uracil correction, and, thus, error-free DNA repair. In contrast, the binding of FAM72A with UNG2 antagonizes UNG2 activity and causes UNG2 degradation in B cells, leading to increased levels of genome-wide deoxyuracils and, therefore, mediating increased levels of U•G mispairs that engage in mutagenic mismatch repair, promoting the error-prone processing of activation-induced cytidine deaminase (AID)-induced deoxyuracils [27,28]. Thus, FAM72A bridges BER and mismatch repair in order to modulate antibody diversification during B cell and antibody maturation [27,28]. Overall, an increased FAM72A level could lead to reduced UNG2 levels and could thus shift the balance of appropriate mutagenic DNA repair, therefore making the cells more susceptible to mutations, with possible effects on tumor development [24,25,27,28,29].

To understand the possible disruption of the FAM72A-UNG2 interaction, the current investigation conducted an in silico prediction of FAM72A-UNG2 heterodimer–protein interaction and the identification of potential chemicals that interfere with the FAM72A-UNG2 heterodimer protein activity for the potential treatment of cancer.

## 2. Materials and Methods

### 2.1. Data Collection—Sequence and Structure Details

The crystallographic three-dimensional (3D) protein structure of the FAM72A protein (from Protein Data Bank (PDB) data [30]) and UNG2 protein (Gene ID: 7374, isoform-2: NP_550433.1, 313 AAs; 1AKZ_A PDB model; DOI: 10.2210/pdb1akz/pdb [31]) were retrieved from the PDB [32] with a resolution of 1.55 Å. Compound structures were downloaded from the PubChem database [33] as described previously [30,34,35].

### 2.2. Homology Modeling and Protein Structure Validation of UNG2 by Modeller, I-TASSER and AlphaFold

FAM72A 3D protein structure was used from previously designed PDB data [30]. Unfortunately, no suitable UNG2 3D protein structure was available, and the N-terminal UNG2 residues were called an intrinsically disordered region. Thus, the UNG2 protein sequence was checked in the National Center for Biotechnology Information (NCBI) and PDB, and the closest suggested template for a UNG2 3D protein structure model was selected. The UNG2 3D peptide sequence was based on the UNG2 protein sequence (Gene ID: 7374, isoform-2: NP_550433.1, 313 AAs; Uniprot-ID: P13051) and the 1AKZ_A PDB model (DOI: 10.2210/pdb1akz/pdb) [31] was selected as the template. The obtained template for the N-terminal UNG2 3D peptide structure model (AA 1-313) was then forwarded for UNG2 3D peptide structure modeling with I-TASSER [36,37] and Modeller v9.20 [38,39] software, and Chimera software was used as a graphical interface as described previously [34,35,39]. For comparison, we also applied the UNG2 protein sequence to the state-of-the-art machine learning method, AlphaFold ((https://alphafold.ebi.ac.uk/), accessed on 3 November 2021) [40,41].

### 2.3. Intrinsically Disordered Region in UNG2 (AA 1–92)

The N-terminal regulatory region of UNG2 is described as an intrinsically disordered region by several groups [16,17,42]. In continuation, JPred4 [43] was used for additional secondary structure prediction. A search algorithm and sequence weighting method against the given UNG2 protein sequence was applied with default parameters (hidden Markov model (HMM) and BLOSUM filter). The UNG2 AA composition was calculated to identify and justify the AA residues promoting structured or unstructured regions in the UNG2 protein. Modeled structures were visualized using Chimera software as the graphical interface to check the core, rim, and buried regions, as described previously [30,34,35]. 

### 2.4. Molecular Docking of FAM72A Protein and UNG2 Peptide (AA 1–45) by HPEPDOCK

Docking interactions for the FAM72A protein with the modeled UNG2 peptide (AA 1–45) as a heterodimer were performed by HPEPDOCK (default parameters were applied) [44]. The FAM72A monomer was exported as a PDB file, whereas the UNG2 peptide was submitted as a FASTA-formatted AA sequence (AA 1–45), and MODPEP and MDOCK were applied for the fine adjustments of FAM72A-UNG2 interactions [44]. Modeled structures were visualized using Chimera software as a graphical interface, as described previously [30,34,35].

### 2.5. Molecular Mechanics/Generalized Born Surface Area (MM/GBSA) Calculation

The molecular mechanics/generalized Born surface area (MM/GBSA) free energy decomposition per AA residue in protein–protein interactions was predicted on the FAM72A protein and UNG2 (AA 1–45) peptide heterodimer [45]. Hawkdock calculated the free binding energy for the key AA residues in the protein–protein interfaces based on the Amber16 force field [46].

### 2.6. Carbon Distribution (CARd) Analysis

The protein carbon distribution (CARd) analysis was performed to validate the specific FAM72A-UNG2 interaction sites using our recently described algorithm [35,47].

### 2.7. Amino Acid-Specific Mutations in FAM72A Protein-UNG2 (AA 1–45) Peptide Heterodimer (FAM72A F104A, F104R, F104N, F104G, and F104S)

A site-specific mutagenic approach was enabled to check the hot spot residues in the FAM72A protein and UNG2 (AA 1–45) peptide heterodimer interaction. AA modifications in FAM72A (F104A, F104R, F104N, F104G, and F104S) and the effect on the conformational stability of the FAM72A protein and UNG2 (AA 1–45) peptide heterodimer complex were investigated by the BIOVIA Discovery Studio software (Dassault Systems; Waltham, MA, USA) as described previously [48,49].

### 2.8. Molecular Dynamics Simulation by GROMACS

The starting coordinates of FAM72A and UNG2 were taken from the modeled structures, as described in Section 2.2. GROMOS96 43a1 force field was used in this study. Hydrogens were added to the protein molecules by using the pdb2gmx application in GROMACS (2019.2). Then, protein molecules were placed in a cubic simulation box (default parameters). A simple point charge water model was used for the solvation simulation box. To neutralize the system, Na^+^ and Cl^−^ ions were added to the simulation box. The structure was relaxed through a process called energy minimization (EM). Subsequently, energy-minimized structures were used for the system equilibration performed under constant NVT and NPT (number, volume, temperature, and pressure) ensembles. The production run was carried out using the NPT ensemble for 50 ns with a time step of 2 fs at a constant temperature of 300 K and 1 bar pressure. Simulation trajectories were visualized using Visual Molecular Dynamics (VMD) 1.9.4a42. Analysis of features, including root mean square deviation (RMSD), root mean square fluctuation (RMSF), and radius of gyration (Rg), were performed using GROMACS (2019) tools [50]. RMSD is the most commonly used metric, in which, the root mean square distance between corresponding residues is calculated [51,52]. Since the RMSD can weight the distances between all residue pairs equally, a small number of local structural deviations can result in high RMSD, even when the global topologies of compared structures are similar. The average RMSD of randomly related proteins depends on the length of compared structures, rendering the absolute magnitude of RMSD meaningless [53]. The RMSD computes the average distance between the backbone atoms of starting structure (reference structure) with simulated structures (frame by frame) when superimposed. The RMSF computes fluctuations (standard deviation) of atomic positions of each AA residue in the trajectory. The RMSD and RMSF were calculated for 50 ns using GROMACS (2019) [50] for the FAM72A-UNG2 heterodimer with the UNG2 (AA 1–45) peptide and FAM72A protein (wildtype [wt], W125A, W125R, F104A, F104R, F104G, F104N, and F104S) [50]. The trajectory files resulting from the molecular dynamics simulation were computed as RMSD, RMSF, and Rg, and were plotted by Grace (“GRaphing, Advanced Computation and Exploration of data”; a WYSIWYG 2D graph plotting tool for Unix operating systems).

### 2.9. Virtual Screening for Lead Molecule Identification against FAM72A Protein and UNG2 (AA 1–45) Peptide Mono/Heterodimer

The MTiOpenScreen [54], along with Autodock vina [55] and iScreen [56] databases, were applied for chemical library virtual screening and de novo drug design. Additionally, pharmacophore analysis was performed using the PharmMapper server [57] to detect the basic pharmacophore group of select chemicals for docking analysis. Further application of COACH, TM-SITE, S-SITE, COFACTOR, and ConCavity approaches [30,34,35] provided potential ligand-binding sites of the 3D FAM72A-UNG protein heterodimer or the FAM72A protein monomer structure model (with refinement by ModRefiner [58]), with potential molecules based on a BioLiP [59]. 

### 2.10. Molecular Docking of FAM72A Protein and UNG2 (AA 1–45) Peptide Heterodimer and FAM72A Monomer with Small Chemical Molecules

Further molecular docking studies have been undertaken in order to gain further insights into the possible FAM72A-UNG2 binding interference by molecules newly identified by protein–ligand binding site prediction and to understand their mechanisms of interaction. Identified molecules obtained by the virtual screening were docked onto the FAM72A protein and UNG2 (AA 1–45) peptide heterodimer and/or FAM72A monomer using Schrödinger to depict binding mode and calculate binding energy [60,61]. FAM72A and UNG2 3D protein structures were prepared using the protein preparation wizard panel of the Schrödinger software package (Schrödinger, LLC, New York, NY, USA). The 3D protein crystal structures of FAM72A and UNG2 were transferred to the workspace and pre-processed, and missing loops were filled [62]. Water molecules were removed from the ligand-binding domain. H-bonds were optimized using the hydrogen bond optimizer, and the FAM72A and UNG2 protein structures were moved to the minimization process to minimize the energy in order to confirm the lowest energy conformational structure [63]. Default parameters were used for the molecular docking process, applying the Glide 4.0 XP extra precision module of the Schrödinger software package (Schrödinger, LLC). The binding affinity with FAM72A was calculated for each chemical compound and ranked by the scoring function. Modeled structures were visualized using the same Chimera software as the graphical interface, as described previously [30,34,35,62].

## 3. Results and Discussion

### 3.1. Homology Modeling and Protein Structure Validation of UNG2 by Modeller, I-TASSER and AlphaFold

**Figure 1:**
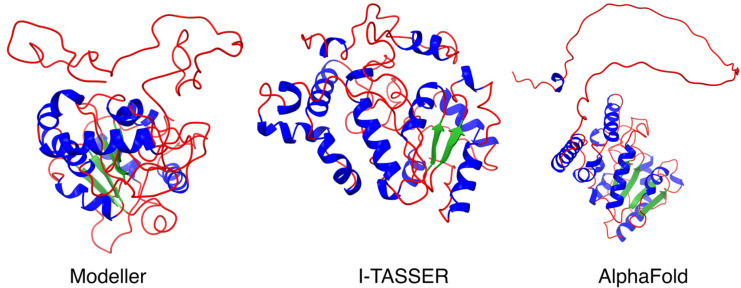
Predicted comparative 3D protein structure analysis of full-length UNG2 protein, including N-terminal UNG2 (AA 1–92) (**Left**): Modeler modeled full-length UNG2 3D protein structure output (using human UNG2 sequence from UniProtKB (P13051) and PDB template 1AKZ) implying a conformation suitable for N-terminal UNG2 protein binding to chromatin; (**Center**): I-TASSER modeled full-length UNG2 3D protein structure output implying a conformation suitable for the binding of the N-terminal UNG2 protein to its catalytic site; (**Right**): AlphaFold output of full length UNG2 (using human UNG2 sequence from UniProtKB (P13051). All three protein structure prediction approaches revealed the N-terminal loop as a protruding thread-like disordered region suitable for interactions with multiple protein binding partners and for molecular crowding. Secondary structure color code: α-helix in blue, β-sheet in green, and loop in red.

The UNG2 protein can be functionally divided into two domains: an N-terminal regulatory region (AA 1–92) and a C-terminal catalytic region (AA 93–313). The disordered N-terminal region has been identified as interacting with several proteins, including proliferating cell nuclear antigen (PCNA) and replication protein A (RPA) (both found at DNA replication forks), as well as with FAM72A [2,16,17,18,26,27]. To further enlighten the FAM72A-UNG2 interaction, we investigated the N-terminus of UNG2 (AA 1–92), applying the Modeller, I-TASSER, and AlphaFold protein structure prediction analysis programs. Our predicted comparative structure analysis revealed a long protruding thread-like disordered N-terminal loop (AA 1–92) required to gather and catch more targets for molecular crowding (Figure 1). 

### 3.2. Intrinsically Disordered Region in N-Terminal UNG2 (AA 1–92)

**Figure 2:**
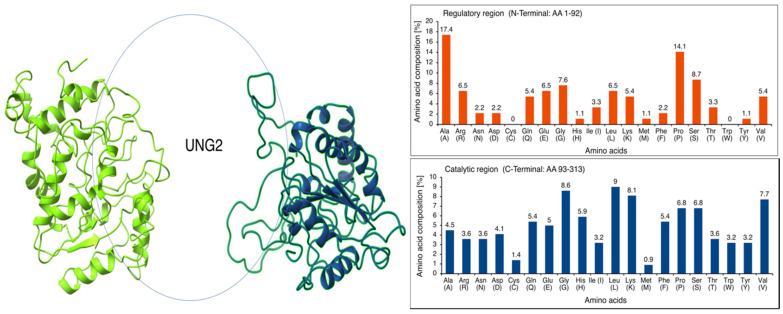
(**Left**): The intrinsically disordered proteins (IDP) regions in the I-TASSER- and Modeler-based predicted models is circled. Left-hand side: I-TASSER output; right-hand side: Modeler output. A similar N-terminal IDP was obtained with AlphaFold (Figure 1). (**Right**): AA composition was calculated for AA residue enrichment in promoting ordered or disordered regions in UNG2. The AA composition of UNG2 analysis clearly shows the abundance of disorder-promoting AAs Ala, Pro, Ser, and Gly within the N-terminal regulatory region, while the catalytic region is enriched by hydrophobic order-promoting AA residues, including Leu, Val, Ile, and Trp. Evidently, Cys and Trp are not available for a foldable secondary structure formation as linker residues form in the N-terminus of UNG2. The plot shows biases in AA composition at N-terminal residues and explains the importance of sulfur-containing AAs and tryptophan at hydrophobic cores for protein rigidity. The absence of AAs, such as Cys and Trp, has been recognized by evolutionary studies of protein plasticity and disordered protein regions in previous studies.

Intrinsically disordered proteins (IDPs) execute various functions in all kinds of cellular processes [64,65,66]. The N-terminal regulatory domain of UNG2 possesses such an unstructured regional IDP motif (AA 1–92). In general, the absence of a hydrophobic core is probably the reason for an unstructured region, whereby hydrophilic AAs may dominate in number. AAs have been classified as order-promoting (Asn, Cys, Ile, Leu, Phe, Trp, Tyr, and Val) and disorder-promoting (Ala, Arg, Gln, Glu, Gly, Lys, Pro, and Ser) [65,67]. The calculated AA composition revealed an abundance of disorder-promoting Ala, Pro, Ser, and Gly in the N-terminal regulatory region, which is very flexible in moving and orienting the N-terminus of UNG2. In contrast, the catalytic region is full of hydrophobic order-promoting AA residues, including Leu, Val, Ile, and Trp. Evidently, Cys and Trp are not available for a foldable secondary structure formation, as linker residues form in the N-terminus of the UNG2. The plot shows the biases in AA composition at N-terminal residues and explains the importance of sulfur-containing AAs and tryptophan at hydrophobic cores for protein rigidity. The absence of AAs, such as Cys and Trp, has been recognized by evolutionary studies about protein plasticity and disordered protein regions in previous studies (Figure 2) [66,68,69,70].

### 3.3. FAM72A-UNG2 Interaction and Molecular Docking Study of FAM72A Protein and UNG2 (AA 1–45) Peptide by HPEPDOCK

**Figure 3:**
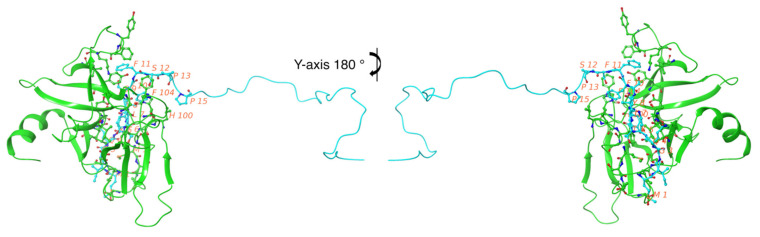
FAM72A-UNG2 (AA 1–45) interactions. FAM72A = chain-A is shown in green and UNG2 (AA 1–45) = chain-B is shown in blue. The image on the right-hand side is a *y*-axis 180° of the FAM72A-UNG2 interaction to illustrate the interaction clearly. Key interacting AA residues are labeled in red. The prevalence rate of interface residues on the FAM72A-UNG2 interaction has been depicted by molecular rendering (cartoon model). Hydrophobicity is the major phenomenon of the FAM72A protein and UNG2 peptide interaction.

Our molecular docking study evaluated the molecular forces responsible for specific biomolecular FAM72A-UNG2 interactions. The FAM72A monomer was exported as a PDB file (FAM72A 3D protein structure was used from previously designed PDB data [30]), whereas the UNG2 (AA 1–45) peptide was submitted as a FASTA-formatted AA sequence (AA 1–45). The UNG2 (AA 1–45) peptide was used because these AAs appeared to be the pivotal interacting AAs [2,16,17,18,26]. The docked structure was analyzed for specific AAs contributing to the FAM72A protein and UNG2 peptide interactions. Mostly, electrostatic forces dominate other forces. Hydrogen bonding is less preferred for the FAM72A-UNG2 association because the side chain (UNG2; chain-B) moves along the diagonal portion of the other FAM72A protein (chain-A). Due to the lack of a proper quaternary structure in the N-terminal UNG2 region, the UNG2 peptide prefers surface AA residues (such as AAs 5, 7, 8, 10, 11, 12, 13, and 15) in order to make connections and to increase the prevalence rate of interactions (Figure 3).

### 3.4. Free Binding Energy Prediction on FAM72A Protein and UNG2 (AA 1–45) Peptide Heterodimer

**Figure 4:**
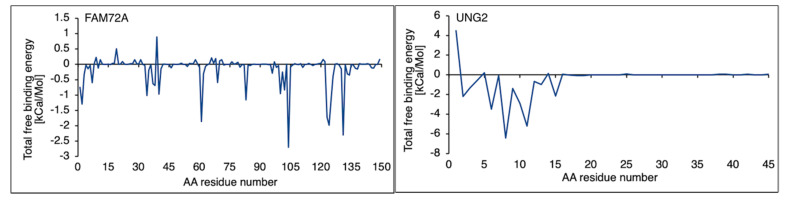
Free binding energy was calculated for the FAM72A protein and UNG2 (AA 1–45) peptide heterodimer interaction. Specific AA residue–residue contact energy calculated the FAM72A-UNG2 (AA 1–45) interaction. Key AA residues were identified in FAM72A (2, 37–40, 61, 83, 101–104, 123–126, and 131, respectively), while AA F104 was identified as a pivotal AA with the highest binding energy contacting the Ser12 and Pro13 AAs of UNG. The UNG2 (AA 1–45) peptide has been verified with only a few AAs accountable for binding contribution (AAs 2, 5, 8, 11, and 15, respectively).

An MM/GBSA prediction is imposed on the free binding energy calculation in the FAM72A protein and UNG2 (AA 1–45) peptide heterodimer. The MM/GBSA analysis offered a breakthrough regarding catalytic AAs in the FAM72A protein and UNG2 (AA 1–45) peptide heterodimer. AA residue–residue contacts in the FAM72A-UNG2 heterodimer were calculated in terms of free binding energy, considering van der Waals forces, electrostatic energy, solvent accessible surface areas, and polar and non-polar energies. Binding affinity values were plotted as a scatter plot indicating key AA residues in FAM72A (2, 37–40, 61, 83, 101–104, 123–126, and 131, respectively), while AA F104 was identified as pivotal AA. In proportion, the UNG2 (AA 1–45) peptide has been verified with only a few AAs accountable for binding contribution (AAs 2, 5, 8, 11, and 15, respectively) (Figure 4).

### 3.5. AA-Specific Mutations in the FWMF Motif (AA 101–104) of FAM72A Affecting the FAM72A Protein and UNG2 (AA 1–45) Peptide Heterodimer Binding

**Figure 5:**
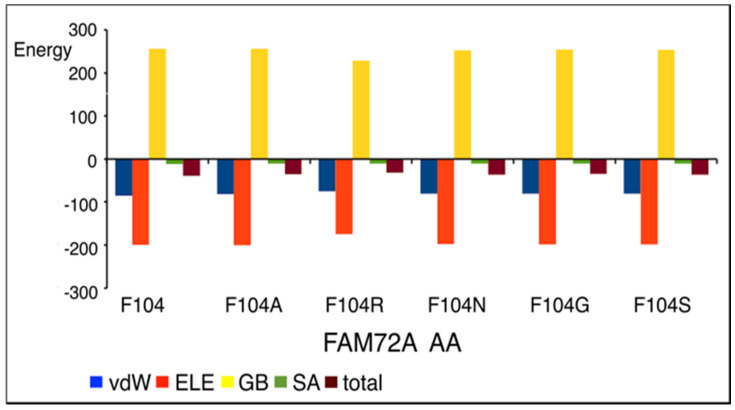
FWMF motif (AA 101–104)-specific mutation-dependent FAM72A protein and UNG2 (AA 1–45) peptide heterodimer free binding energy (energy in [kcal/mol]). FAM72A mutations at AA F104 phenylalanine (F104 → 104A, F104R, F104N, F104G, and F104S). A difference in total free binding energy was observed individually at the FWMF motif (AA 101–104)-specific mutations, with F104R mutation showing the highest effect on energy-dependent conformational change affecting FAM72A-UNG2 binding. The FAM72A protein and UNG2 (AA 1–45) peptide heterodimer free binding energy was calculated by the MM/GBSA approach. ELE, electrostatic; GB, polar accessible (a grid-based surface GB model is used for estimating the polar component of solvation free energy, coupled with water and atomic radii); SA, solvent area; total, sum of total energy; vdW, van der Waals; MM/GBSA, molecular mechanics/generalized Born surface area.

**Figure 6:**
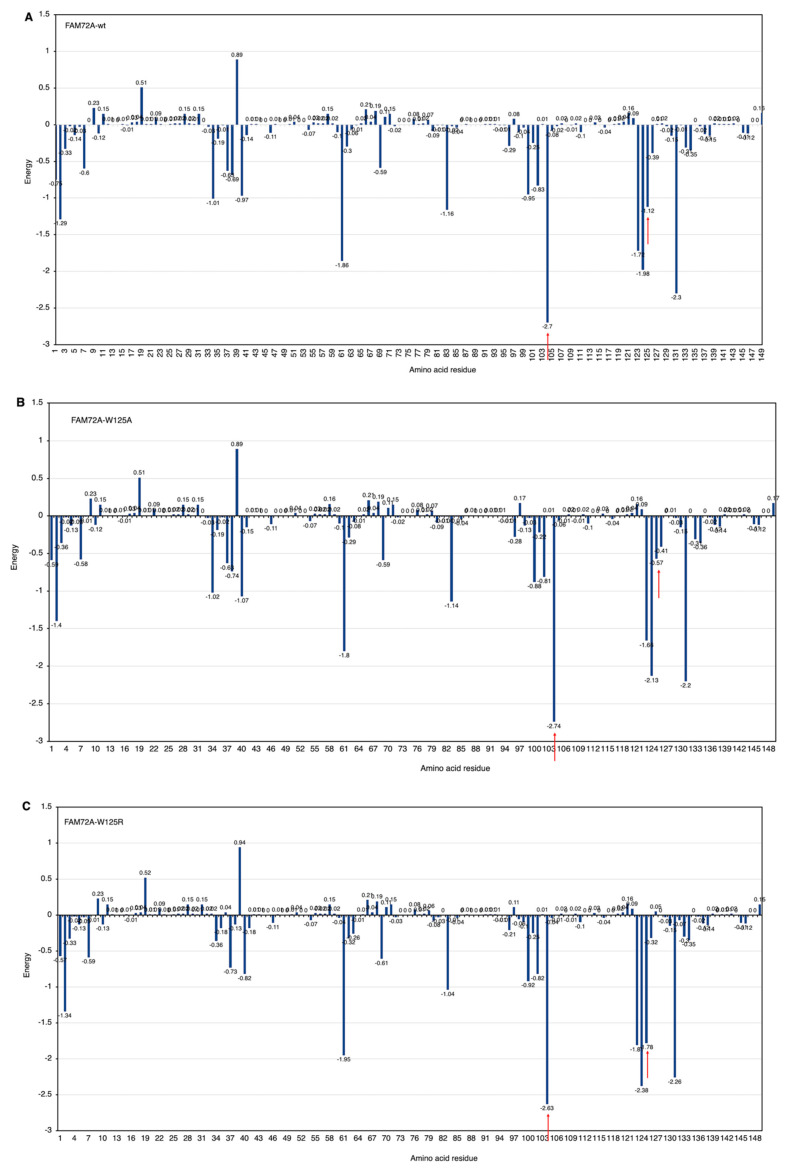

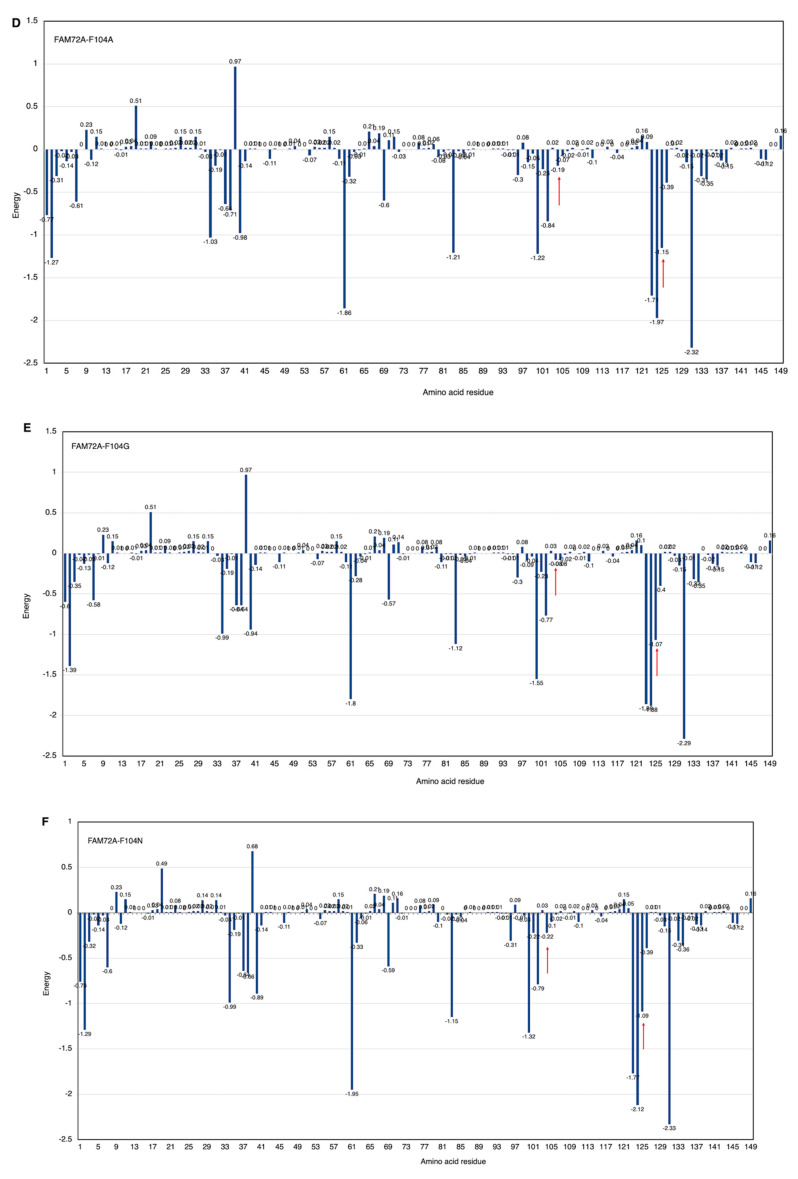

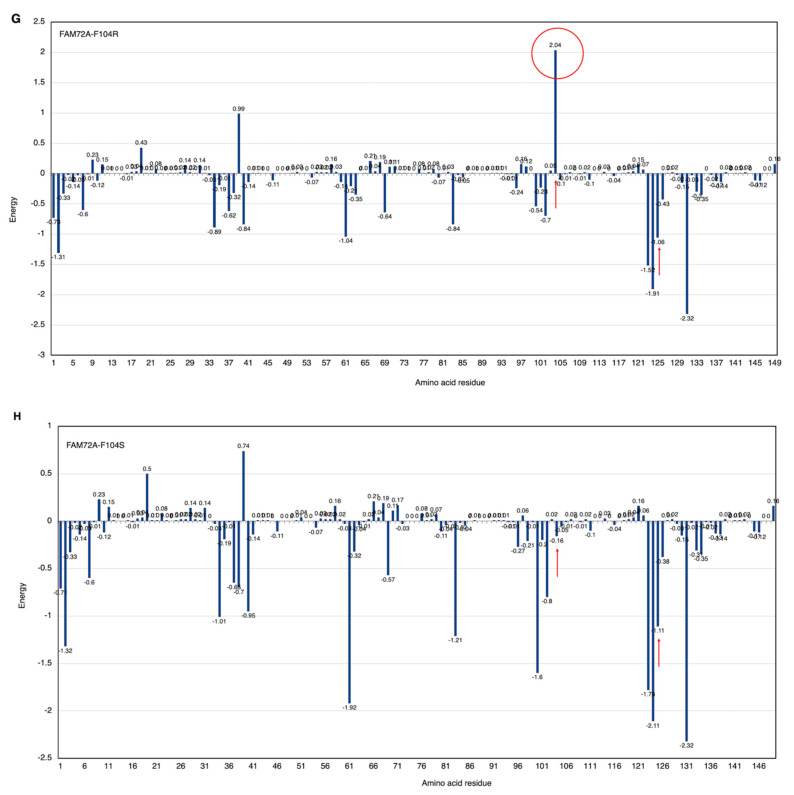
(**A**–**H**) FWMF motif (AA 101–104)-specific mutation-dependent FAM72A protein and UNG2 (AA 1–45) peptide heterodimer free binding energy. FAM72A mutations at AA F104 phenylalanine (F104 → F104A, F104R, F104N, F104G, and F104S). The essential AA residues in FAM72A-wt and UNG2 interaction are the 2, 37–40, 61, 83, 104, 123–126, and 131 AAs of FAM72A. The highest binding energy (kcal/mol) goes to FAM72A’s AA F104 within the FWMF motif (AA 101–104). Positive values indicate low binding energies and weak interactions, whereas more negative values indicate stronger interactions. Values around 0 indicate moderate interactions between FAM72A and UNG2. A change in the FWMF motif from wt F104 to F104R shows the largest effect, turning the binding energy from negative (−2.7; strong binding/hydrophobic core) to positive (+2.04; strong binding/hydrogen bonding).

Site-directed specific mutations in the FWMF motif (AA 101–104) of FAM72A were used (F104A, F104R, F104N, F104G, and F104S) to evaluate the rigidity and flexibility of the interface in the FAM72A protein and UNG2 (AA 1–45) peptide heterodimer binding (Figure 5 and Figure 6).

We modeled the FAM72A and UNG2 (AA 1–45) peptide and the interaction of FAM72A with the UNG2 (AA 1–45) peptide. The FWMF motif (AA 101–104) appears to be key for the FAM72A protein structure and its binding to UNG2 (Figure 6). A mutation in the FWMF motif from wt F104 to F104R had the largest effect, turning the binding energy from negative (strong binding/hydrophobic core) to positive (strong binding/hydrogen bonding).

### 3.6. Molecular Dynamic Simulation by GROMACS Validates AA-specific Mutations in the FWMF Motif (AA 101–104) of FAM72A Affecting FAM72A-UNG2 Heterodimer Binding

**Figure 7:**
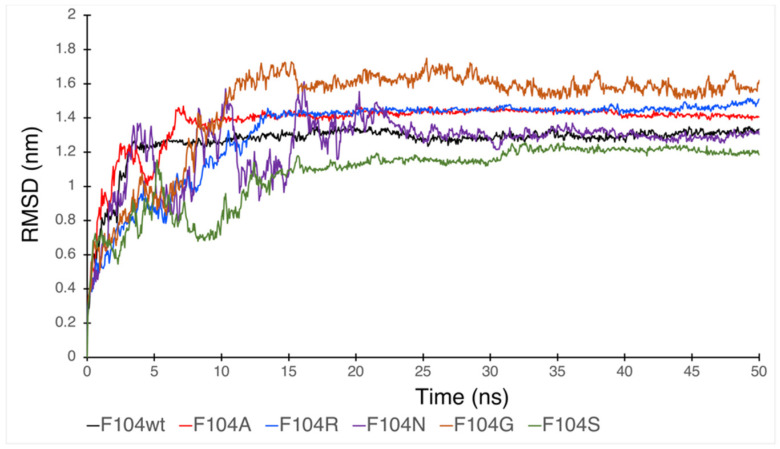
RMSD plot showing the effect of FAM72A mutations at wt AA F104 phenylalanine (F104 → F104A, F104R, F104N, F104G, and F104S) within the FWMF motif (AA 101–104) on the dynamic nature of the FAM72A protein and UNG2 (AA 1–45) peptide heterodimer binding.

**Figure 8:**
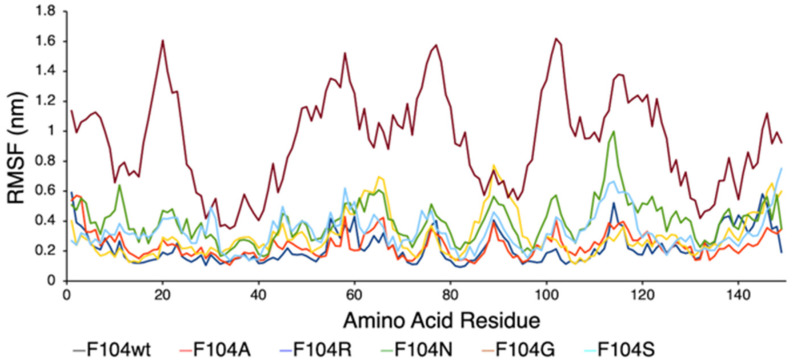
RMSF plot showing the effect of FAM72A mutations at wt AA F104 phenylalanine (F104 → F104A, F104R, F104N, F104G, and F104S) within the FWMF motif (AA 101–104) on the dynamic nature of the FAM72A protein and UNG2 (AA 1–45) peptide heterodimer binding.

**Figure 9:**
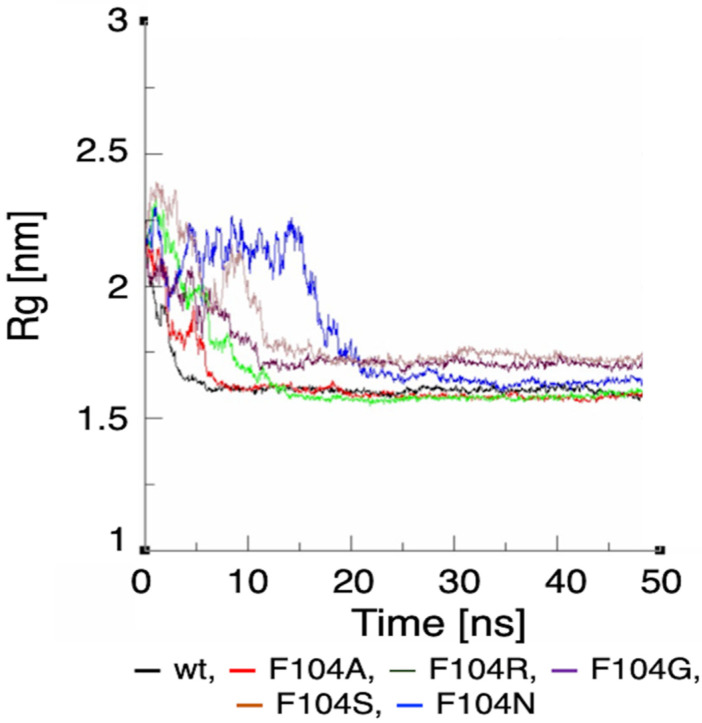
Rg plot showing the effect of FAM72A mutations at AA F104 phenylalanine (F104 → F104A, F104R, F104N, F104G, and F104S) within the FWMF motif (AA 101–104) on the dynamic nature of the FAM72A protein and UNG2 (AA 1–45) peptide heterodimer binding.

Since phenylalanine F104 appeared to be the key AA within the FWMF motif (AA 101–104) at the interface of the FAM72A-UNG2 interaction, we further investigated the effect of FAM72A mutations at wt AA F104 phenylalanine (F104 → F104A, F104R, F104N, F104G, and F104S) within the FWMF motif (AA 101–104) on the dynamic nature of FAM72A-UNG2 binding. Dynamic conformation changes in FAM72A-UNG2 (AA 1–45) binding were simulated by GROMACS and plotted by Grace (Figure 7, Figure 8 and Figure 9). 

We assessed the effect of F104 mutations in FAM72A on the dynamic confirmation changes, stability, and rigidity of core and buried regions. Trajectories recorded up to 50 ns were plotted as RMSD, RMSF, and Rg, respectively. Figure 7, Figure 8 and Figure 9 show the effect of these mutations on protein backbone changes in mutated FAM72A and signifies the pivotal role of this FWMF motif (AA 101–104) for the FAM72A-UNG2 interaction. These data confirm the FWMF motif as a suitable target to interfere with FAM72A-UNG2 signaling pathways.

### 3.7. CARd Analysis

**Figure 10:**
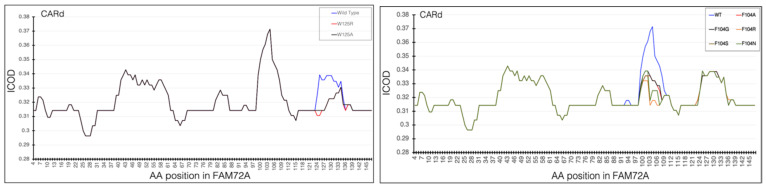
CARd analysis showing the effect of FAM72A mutations at the AA tryptophan Trp W125 (W125 → W125A and W125R) and F104 phenylalanine (F104 → F104A, F104R, F104N, F104G, and F104S; within the FWMF motif (AA 101–104)) on the dynamic nature of the FAM72A protein and UNG2 (AA 1–45) peptide heterodimer binding. CARd analysis confirmed the FAM72A-UNG2 interaction and the effect of the various FAM72A mutations on it, as carbon governs cohesiveness at the active site in proteins. Accordingly, internal carbon optimized domains (ICODs) of FAM72Awt and UNG2 (AA 1–45) interaction validate a strong overlap of FAM72A-UNG2 binding. In particular, mutations in FAM72A turn the ICODs of FAM72A at AAs 100–104 and 125–135 into non-ICODs, encouraging a less favorable and weaker FAM72A_mut_-UNG2 induced-fit-binding than that of FAM72A_wt_-UNG2. F104A, F104G: change from hydrophobic (W) and carbon-rich (W) to hydrophobic and carbon-low; F104R: change from hydrophobic (F) and carbon-rich (F) to hydrophilic, carbon-rich, and basic AA); F104S, F104N: change from hydrophobic (F) and carbon-rich (F) to hydrophilic, carbon-low, and polar AA. CARd reveals that F104 tends to switch to F104R > F104S > F104N > F104G > F104A. Code information: F104: uuu, A104: gcu, G104: ggu, S104: ucu, N104: aau, R104: cgu.

Knowing that AAs Trp/W125 and F104 in FAM72A are important for the FAM72A-UNG2 interaction, we changed wt FAM72A-W125 (FAM72A-wt) to FAM72A-W125A or W125R, and wt FAM72A-F104 to F104A, F104R, F104N, F104G, and F104S, respectively, and performed CARd analysis (Figure 10).

Internal carbon optimized domains (ICODs) of the FAM72A_wt_-UNG2 (AA 1–45) heterodimer confirmed the FAM72A_wt_-UNG2 (AA 1–45) interaction and the effect of the various FAM72A mutations on it, as carbon governs cohesiveness at the active site in proteins. Accordingly, ICODs of the FAM72A_wt_-UNG2 (AA 1–45) interaction validate a strong overlap of FAM72A-UNG2 binding. Particularly, mutations in FAM72A turn the ICODs of FAM72A at AAs 100–104 and 125–135 into non-ICODs, encouraging a less favorable and weaker FAM72A_mut_-UNG2 induced-fit-binding than that of FAM72A_wt_-UNG2. Based on CARd analysis, F104 tends to switch to F104R > F104S > F104N > F104G > F104A. The likelihood of the F104 (uuu) mutation to S104: ucu appears to be highest, as only one nucleotide would be changed compared with the others (F104R: cgu, F104N: aau, F104G: ggu, and F104A: gcu).

### 3.8. Lead Discovery and Chemical Docking—Interference with FAM72A-UNG2 Interaction and Activity

**Figure 11:**
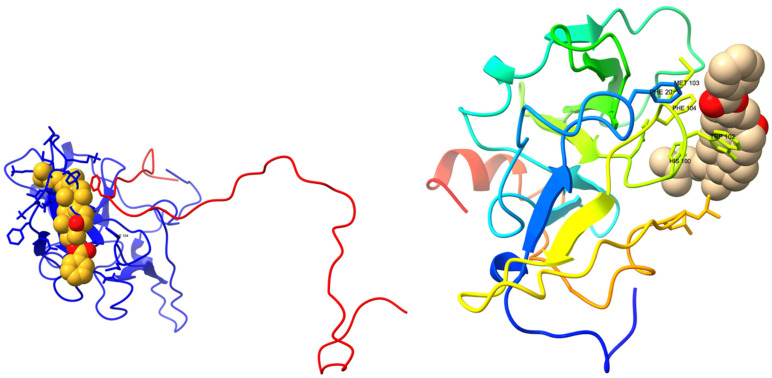

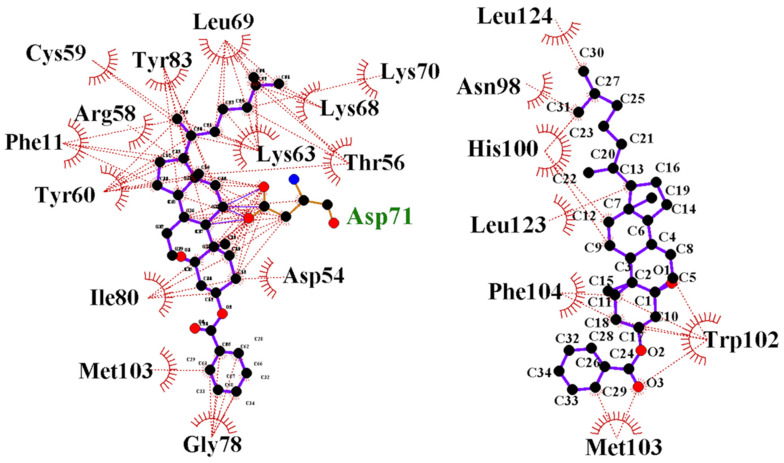
Interaction of the FAM72Awt-UNG2 (AA 1–45) heterodimer with withaferin B, another withanolide analog. (**Top left-hand side**): Schematic 3D interaction of FAM72A protein and UNG2 (AA 1–45) peptide heterodimer (cartoon model) with withaferin B (space-fill model) is shown. (**Top right-hand side**): FAM72A protein monomer (cartoon model) with withaferin B (space-fill model) is shown. (**Bottom left-hand side**): LIGPLOT output showing the interaction between the FAM72Awt-UNG2 (AA 1–45) heterodimer and withaferin B. Green Asp71 indicates hydrogen bonding. (**Bottom right-hand side**): LIGPLOT output showing the interaction of FAM72Awt monomer with withaferin B. The binding affinity was calculated as −1.868 kCal/Mol. Withaferin B, PubChem compound ID (CID): 11113907, molecular formula: C_34_H_50_O_3_, molecular weight: 506.8 g/mol. IUPAC Name: [(1S,2R,5S,7S,9R,11S,12S,15R,16R)-2,16-dimethyl-15-[(2R)-6-methylheptan-2-yl]-8-oxapentacyclo [9.7.0.0^2,7^.^07,9^.0^12,16^] octadecan-5-yl] benzoate. UNG2′ AAs Tyr(Y)8 and Phe(F)11 are the most important AAs for the interaction with the FAM72A FWMF (AAs 101–104) motif. Withaferin B binds to the FAM72A-UNG2 heterodimer’s interface at the FWMF motif and interacts strongly with both FAM72A and UNG2. Our data show that withaferin B could probably bind to the FAM72A-UNG2 heterodimer using electrostatic interactions and hydrophobic contacts via FAM72A’ AAs Y60, T56, C59, and M103 and hydrogen bonding with FAM72A’ D71. Moreover, withaferin B could probably bind to the FAM72A-UNG2 heterodimer using hydrophobic contacts via UNG2′ AA F11 to disrupt the stability of FAM72A-UNG2 chain attachment and, thus, to inhibit the formation of active FAM72A-UNG2 protein complexes. As a result, FAM72A-UNG2 cell signaling could be turned off.

We performed a virtual high-throughput screening to detect a potential lead interference with the FAM72A-UNG2 interaction [71,72,73]. Binding scores, along with drug-likeness and pharmacophore properties, were considered [35,74,75,76,77,78]. The MTiOpen Screen required Autodock vina to exercise the docking of chemical libraries, scoring, and ensemble analysis [54,55]. The virtual screening suggested 100 compounds, and predicted binding energies (kcal/mol) were considered to filter the hits for the selection of the “best” hit for optimization in order to identify the promising lead compound. Based on predicted binding energies, we identified withaferin B (PubChem CID: 11113907) as the “best” hit (binding energy −0.5 kcal/mol) molecule that could potentially interfere with the FAM72A-UNG2 interaction (Figure 11). In the lead generation, the Glide XP docking analysis showed a strong binding affinity of −1.868 kcal/mol at the FAM72A-UNG2 interference site. Active AA residues contributing to the interaction were visualized by LIGPLOT (Figure 11).

Interestingly, withaferin B has structural similarities with withaferin A. Both compounds are withanolide analogues (derived from *Withania somnifera* (Indian ginseng)) and contain an oxapentacyclo moiety. Besides, in their central moieties, withaferin B contains octadecan-5-yl, whereas withaferin A contains octadec-4-en-3-one. Of note, withaferin A has been reported to be a potential anti-cancer molecule that can inhibit cell proliferation, cell migration, and cell invasion [79,80,81,82,83,84,85,86]. Similarly, withaferin B, bound to the FAM72A-UNG2 heterodimer, could possibly block FAM72A-UNG2 signaling pathways in cancer cells [26,29]; however, thus far, the biological and therapeutic properties of withaferin B remain unknown.

## 4. Conclusions

Accumulating evidence indicates the involvement of FAM72A in tumorigenesis [24,25,26,29,87,88]. Elevated FAM72A causes reduced UNG2 levels, eventually leading to new mutations [24,25,27,28,29]. Our data pave the way for new investigative experimental approaches to validate the prevention of cancer by interfering with the FAM72A-UNG2 signaling pathways using withaferin B. Withaferin B is a potential candidate for future investigations in the interference with genome stability, centromere formation, and genome editing, and on potential therapeutic strategies for the treatment of cancer.

## Data Availability

The data presented in this study are available on request from the corresponding authors.
